# Development of simple sequence repeat markers for sugarcane from data mining of expressed sequence tags

**DOI:** 10.3389/fpls.2023.1199210

**Published:** 2023-10-23

**Authors:** Huahao Jiang, Muhammad Waseem, Yong Wang, Sana Basharat, Xia Zhang, Yun Li, Pingwu Liu

**Affiliations:** ^1^ College of Agriculture, Guangxi University, Nanning, China; ^2^ School of Breeding and Multiplication (Sanya Institute of Breeding and Multiplication), Hainan University, Sanya, China; ^3^ School of Tropical Agriculture and Forestry (School of Agriculture and Rural Affairs, School of Rural Revitalization), Hainan University, Haikou, Hainan, China; ^4^ Department of Botany, University of Agriculture Faisalabad, Faisalabad, Pakistan

**Keywords:** sugarcane, plant breeding, simple sequence repeats (SSR), SSR loci, unigene annotation

## Abstract

Sugarcane (*Saccharum* spp. hybrids) is a worldwide acclaimed important agricultural crop used primarily for sugar production and biofuel. Sugarcane’s genetic complexity, aneuploidy, and extreme heterozygosity make it a challenging crop in developing improved varieties. The molecular breeding programs promise to develop nutritionally improved varieties for both direct consumption and commercial application. Therefore, to address these challenges, the development of simple sequence repeats (SSRs) has been proven to be a powerful molecular tool in sugarcane. This study involved the collection of 285216 expressed sequence tags (ESTs) from sugarcane, resulting in 23666 unigenes, including 4547 contigs. Our analysis identified 4120 unigenes containing a total of 4960 SSRs, with the most abundant repeat types being monomeric (44.33%), dimeric (13.10%), and trimeric (39.68%). We further chose 173 primers to analyze the banding pattern in 10 sugarcane accessions by PAGE analysis. Additionally, functional annotation analysis showed that 71.07%, 53.6%, and 10.3% unigenes were annotated by Uniport, GO, and KEGG, respectively. GO annotations and KEGG pathways were distributed across three functional categories: molecular (46.46%), cellular (33.94%), and biological pathways (19.6%). The cluster analysis indicated the formation of four distinct clusters among selected sugarcane accessions, with maximum genetic distance observed among the varieties. We believe that these EST-SSR markers will serve as valuable references for future genetic characterization, species identification, and breeding efforts in sugarcane.

## 1 Introduction

Sugarcane (*Saccharum* spp. hybrids) is a global economic and energy crop, with China ranking third in sugar production. This perennial herb is known for its photosynthetic efficiency, higher biomass accumulation, aneuploid polyploidy (≥8), and genetic heterogeneity (Cordeiro et al., 2003). Conventional sugarcane breeding, primarily by stem cutting is laborious and time-consuming, often taking decades to produce new varieties. The complex genetic background of sugarcane cultivars was derived from interspecific hybridization of *S. spontaneum* L. and *S. officinarum* L. (Garsmeur et al., 2018). The commercial sugarcane cultivars inherit ~70–80% of their chromosomes from *S. officinarum*, ~10–15% from *S. spontaneum*, and the remaining ~5–10% from interspecific recombination (D'hont et al., 1996). The limited introgression in sugarcane breeding has led to a narrow genetic basis in commercial cultivars (Singh et al., 2013).

Simple Sequence Repeats (SSRs) are highly polymorphic short tandem repeats (1 – 6 bp) of nucleotide sequences ubiquitous in the genomes of both eukaryotic and prokaryotic organisms (Tóth et al., 2000). SSRs offer several advantages including transferability between species, co-dominance, minimal expertise, instrumentation dependencies, and reproducibility (Mccouch et al., 2001; Cai et al., 2019). They are widely used in genetic diversity studies (Biswas et al., 2020), population structure analysis (Zalapa et al., 2012), association mapping (Gyawali et al., 2016), and linkage mapping (Sugita et al., 2013). The International Sugarcane Microsatellite Consortium (ISMC) has curated 221 SSR markers in sugarcane cultivar R570 (French Reunion) and Q124 (Australian) (Oliveira et al., 2009). Wu et al. (2019) developed an additional 226 SSR markers using a combined fluorescence-labeled SSR and a high-performance capillary electrophoresis (HPCE) system for parental germplasm of the sugarcane breeding programs in China. Similarly, You et al. (You et al., 2015) successfully employed expressed sequence tag-SSR (EST-SSR) to establish the relationship among 69 varieties of *Colocasia esculenta*. Chen et al. (Chen et al., 2017) characterized 11 varieties of *Lycium* by EST-SSRs. Ukoskit et al. (2019) identified 185 EST-SSRs in cultivated sugarcane “Phil6607” and *S. spontaneum* “S6”. Recently, Xiao et al. (Xiao et al., 2020) identified 46,043 SSRs in the diverse panel of sugarcane (22 accessions).

Genome sequencing revolutionized the discovery and application of SSRs in various plant species, including sugarcane. The release of the *S. spontaneum* genome in 2018 (Zhang et al., 2018) has provided a valuable resource for sugarcane cultivar breeders. Previous efforts have yielded a relatively small number of SSR markers in sugarcane, for instance, 351 EST-SSRs were identified from 4085 EST sequences (Singh et al., 2013), 406 EST-SSR markers with 63 were verified as polymorphic (Ul Haq et al., 2016), and 2005 markers were identified from EST sequences with 65.5% showed polymorphism (Oliveira et al., 2009). Therefore, the development of markers to assess the genetic relationships with a comprehensive set of EST information has become an imperative task. In this study, we attempt to screen EST-SSR based on sugarcane unigenes, particularly those associated with functional genes, and assess the genetic diversity among other sugarcane accessions (10 in total) that have been previously overlooked. Additionally, we also investigate the evolutionary relationship between the sugarcane genome to those of sorghum and maize. We believe that these newly developed EST-SSR markers will provide a valuable reference for sugarcane breeding programs and facilitate species screening and identification.

## 2 Materials and methods

### 2.1 Plant materials

A panel of diverse sugarcane accessions including wild type and eight cultivated were sources from Guangxi, Yunnan, Taiwan, and Fujian. These accessions were maintained at Guangxi University, Nanning, China (**Table 1**).

**Table 1:** Information of sugarcane accessions.

Accession Number	Type	Genotypes	Origin	Pedigree
1	Wild	*S. officinarum*	Guangxi	Unknown
2	Wild	*S. spontaneum*	Guangxi	Unknown
3	Cultivated	Yunrui05-782	Yunnan	Hybrid of wild species
4	Cultivated	Yunrui05-767	Yunnan	Hybrid of wild species
5	Cultivated	ROC10	Taiwan	ROC5 × Taitang152
6	Cultivated	ROC22	Taiwan	ROC5 × 69-463
7	Cultivated	Guitang28	Guangxi	CP80-1018 × CP88-2032
8	Cultivated	Guitang32	Guangxi	Yuenong73-204 × CP67-412
9	Cultivated	Funong40	Fujian	Funong93-3406×Yuetang91-976
10	Cultivated	Funong39	Fujian	Yuetang91-976 ×CP84-1198

### 2.2 EST retrieval and mining

The raw EST sequences (approximately 285216) of sugarcane were downloaded from the NCBI (National Center for Biotechnology Information; http://www.ncbi.nlm.nih.gov/dbEST/, on January 14, 2013). The raw sequences were cleaned to remove the poly A (5′ or 3′ end) or poly T stretches using EST-Trimmer software (http://pgrc.ipk-gatersleben.de/misa/download/est_trimmer.pl). Subsequently, we assembled the EST sequences using Contig Assembly Program 3 (CAP3, http://doua.prabi.fr/software/cap3) DNA sequences assembly program, with parameter set as 90% identity and 40 bp overlap.

### 2.3 Identification of SSR motifs and primer pair design

The assembled EST sequences were subjected to a search for SSR motif using the Microsatellite program (MISA; http://pgrc.ipk-gatersleben.de/misa/) with default parameters as follows: 10 for monomeric repeats, 6 for dimeric repeats, and 5 for trimeric, tetrameric, pentanucleotide, and hexameric repeats each. Subsequently, the primer pair was designed in the program Primer 3.0 with the standard criteria as a primer size of 18 to 27 bp and approximately 20 bp, PCR product size of 100 to 300 bp, GC content from 40 – 60%, and melting temperature (Tm) variation from 57 - 63°C.

For each SSR locus, we selected three primer pairs, and the pair yielding the highest-scoring DNA was selected for subsequent SSR marker studies. *In-silico* PCR analysis of the SSR primer pair was performed using MFEprimer3.2.6 (https://mfeprimer3.igenetech.com/) with default parameter setting, except the Tm was set to 50°C (Yu and Zhang, 2011). The primers were synthesized from Sangon Biotech (Shenzhen, China).

### 2.4 Genomics DNA extraction and SSR genotyping

The genomic DNA (gDNA) was extracted from young sugarcane leaves using the cetyltrimethylammonium bromide (CTAB) method. A Nanodrop spectrophotometer (thermos Scientific, USA) was used for gDNA quantification followed by 1% agarose gel electrophoresis for the quality of gDNA. Finally, the DNA was normalized to 10 ng μL^−1^ for PCR amplification. The PCR reaction was performed in a total reaction volume of 10 uL containing 30−50 ng of gDNA, 2.0 μL of 10×Taq buffer (Mg2+), 0.2 mM each of dNTPs, 0.5 μM each forward and reverse primer, and 0.5 U of Taq DNA polymerase (Clontech, Takara, Shanghai). The resulting PCR products, along with a 2000 bp DNA marker, were separated on an 8% polyacrylamide gel through electrophoresis and visualized using silver staining.

SSR genotyping data were recorded as one (band present) and zero (band absent). The Polymorphism Information Content (PIC) values were computed using the following formula:


$$PIC=1-\;\sum_{i=1}^{n}Pɡ2$$

where Pg represents the frequency of a unique genotype if each SSR marker represents a single locus with n SSR genotypes.

The presence and absence of SSR genotyping data were used to construct the phylogenetic tree of 10 sugarcane accessions using the Neighbor-joining (NJ) method based on Nei’s genetic distance with the MEGAX program.

### 2.5 Unigenes annotation in sugarcane and comparison with sorghum and maize

We annotated all the unigenes containing SSRs against Gene Ontology (GO, http://www.geneontology.org) and Kyoto Encyclopedia of Genes and Genomes (KEGG, http://www.genome.jp/kegg/) databases. To assess the conservation of sugarcane unigenes, we conducted BLASTN searches against the sorghum (Z3116) and maize (B73) genomes, using an e-value threshold of -15 for sorghum and -10 for maize. Our selection criteria included a sequence identity of more than 80% and a sequence length exceeding 100bp.

## 3 Results

### 3.1 Distribution of SSR markers

**Figure 1:**
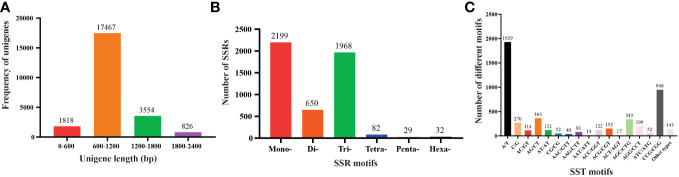
Characteristics of unigenes and SSR from the *Saccharum spontaneum* ESTs **(A)** The number of unigenes based on the number of nucleotides in each, **(B)** the number of SSR motifs in monomeric, dimeric, trimeric, tetrameric, pentameric, and hexameric, and **(C)** the number of different SSR motifs in the unigenes of *Saccharum spontaneum*.

For SSR analysis, a dataset of 285,216 EST sequences retrieved from the NCBI was subjected to quality and redundancy by the CAP3 program. A total of 23666 unigenes sequences including 4547 contigs were generated (**Supplementary Table S1**). The unigenes’ length ranged from a minimum of 101 bp to a maximum length of 4040 bp, with approximately 17467 unigenes’ length varying between 600 to 1200 bases, and 826 unigenes measuring 1800-2400 nucleotides in length (**Figure 1A**). A summary of the sequencing results is presented in **Table 2**. Using the MISA identification tool, we predicted 4120 unigenes containing 4960 SSRs, with a frequency of one SSR/4.43 kb of the available ESTs. Among these sequences, 685 ESTs contained more than one SSR, with 415 being compound SSRs featuring multiple types of repeat motifs.

**Table 2:** Details of ESTs and SSRs identified in sugarcane.

Parameters	Numbers
Total raw EST-sequences	285,216
Contig	4547
Total number of sequences examined	23666
Total size of examined sequences (bp)	22487037
Minimum length of unigenes (bp)	101
Maximum length of unigenes (bp)	4040
Total number of identified SSRs	4960
Number of SSR containing sequences	4120
Number of sequences containing more than 1 SSR	685
Number of SSRs present in compound formation	415
Number of primers designed	3632
Monomeric repeats	2199
Dimeric repeats	650
Trimeric repeats	1968
Tetrameric repeats	82
Pentameric repeats	29
Hexameric repeats	32
Number of motif types	133

**Figure 2:**
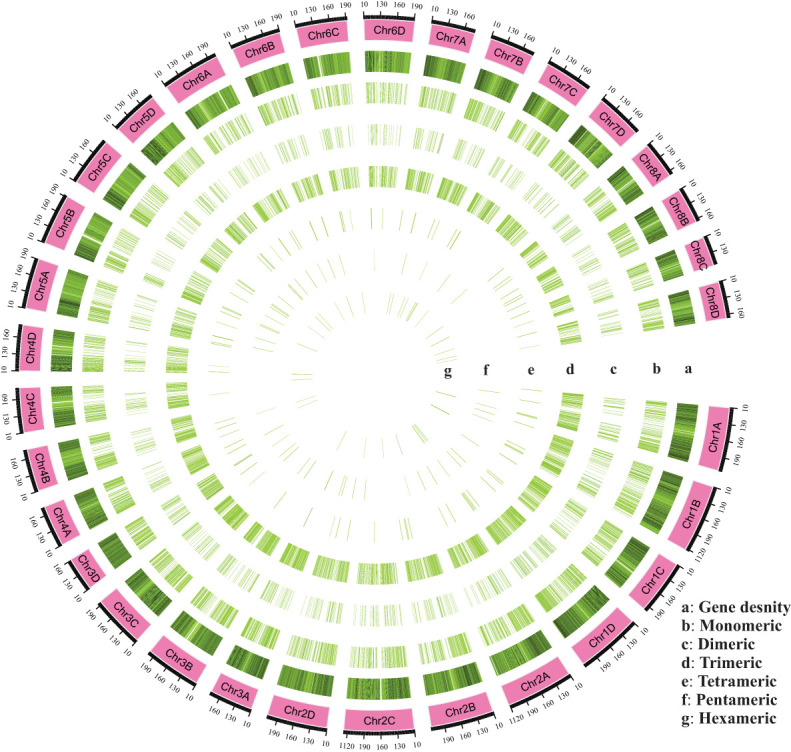
An overall view of the distribution of SSR motifs in the chromosomes (Chr01A-Chr08D) of *Saccharum spontaneum* reference genome. **(A)** Gene density, **(B)** Monomeric, **(C)** Dimeric, **(D)** Trimeric, **(E)** Tetrameric, **(F)** Pentameric, and **(G)** Hexameric.

SSR motifs in the *S. spontaneum* genome were found to be highly frequent within gene regions (**Figure 2** and **Table S1**). Of the 4960 SSR loci, we predicted a total of 133 motif types. Analyzing the abundance of SSR types in sugarcane ESTs, we found that monomeric (44.33%), dimeric (13.10%), and trimeric (39.68%) were the most abundant, followed by tetrameric (1.62%), pentameric (0.58%), and hexameric (0.65%) repeat types (**Figure 1B** and **Table 3**). Furthermore, we observed that the majority of the SSRs had a length of less than 20 bp, with SSRs between 5 – 7 bp and 10 – 12 bp accounting for 75.90% of all the SSRs identified. Additionally, the number of motif types for monomeric, dimeric, trimeric, tetrameric, pentameric, and hexameric were 2, 6, 30, 47, 22, and 26, respectively (**Supplementary Table S2**). The frequency distribution of identified SSRs is shown in **Figure 1C** and **Supplementary Table S3**. Notably, the most copious motif was A/T (1929) followed by CCG/CGG (948), AG/CT (363), AGC/CTG (343), C/G (270), AGG/CCT (199), ACG/CGT (152), ACC/GGT (122), AT/AT (121), AC/GT (114), AAG/CTT (81), ATC/ATG (52), CG/CG (52), AAC/GTT (40), ACT/AGT (17), and AAT/ATT (14). The remaining 67 motif types had a total count of 143 (**Figure 1C** and **Supplementary Table S3**).

**Table 3:** Summary of frequencies of different SSR repeat motif types.

SSR motif	Number of motif types	5	6	7	8	9	10	11	12	13	14	15	>15	total	Proportion (%)
Mono-	2	0	0	0	0	0	729	348	238	148	110	119	507	2199	44.33
Di-	6	0	258	118	79	38	26	17	14	14	10	6	70	650	13.10
Tri-	30	1264	421	177	60	20	11	5	2	3	3	0	2	1968	39.68
Tetra-	47	58	13	3	2	0	0	1	2	1	1	0	1	82	1.65
Penta-	22	21	4	1	1	0	0	1	1	0	0	0	0	29	0.58
Hexa-	26	19	6	5	0	0	1	1	0	0	0	0	0	32	0.65
Total	133	1362	702	304	142	58	767	373	257	166	124	125	580	4960	

**Figure 3:**
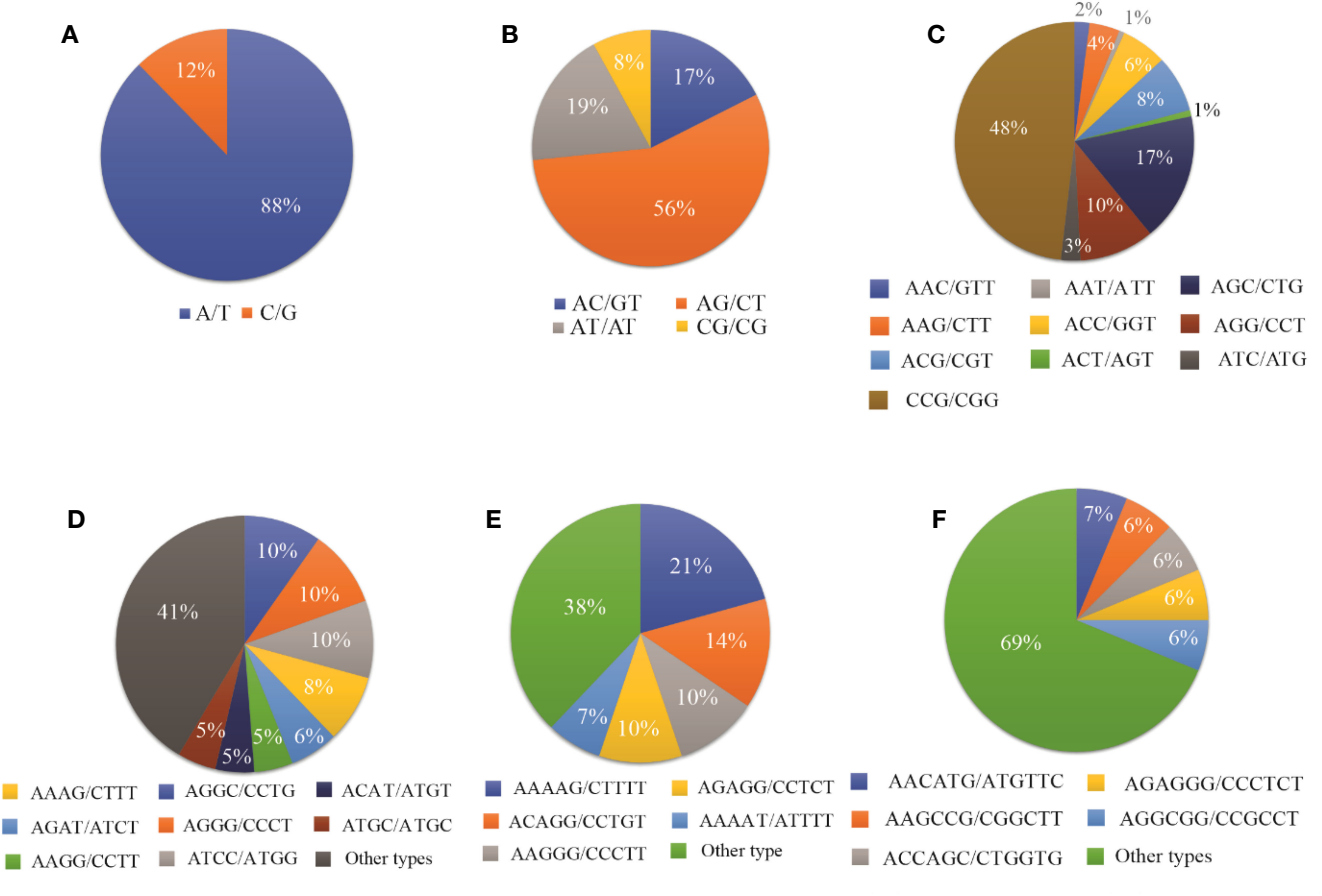
The proportion of different repeat motif types in **(A)** Monomeric, **(B)** Dimeric, **(C)** Trimeric, **(D)** Tetrameric, **(E)** Pentameric, and **(F)** Hexameric.

Among the monomeric repeats, the A/T motif was the most abundant accounting for 88% of all mono repeats (**Figure 3A**). For dimeric repeats, the AG/CT motif dominated, constituting 56% of dimeric repeats, followed by AT/AT (19%), AC/GT (17%), and CG/CG (8%) motif types (**Figure 3B**). In trimeric repeats, CCG/CGG was the most frequent repeat motif, accounting for 48% of trimeric repeats, followed by CGC/CTG (17%), AGG/CCT (10%), ACG/CGT, and ACC/GGT (each at 8%) (**Figure 3C**). Within tetrameric repeats, AGGC/CCTG, AGGG/CCCT, and ATCC/ATGG (10%) were the most abundant repeat motifs, followed by AAAG/CTTT (8%), and AGAT/ATCT (6%). However, 41% of other types of repeats were also detected in tetrameric repeats (**Figure 3D**). Within Pentameric repeats, AAAAG/CTTTT (21%) was the most abundant repeat motif, followed by ACAGG/CCTGT (14%), AAGGG/CCCTT (10%), and AGAGG/CCTCT (10%) (**Figure 3E**). Regarding hexameric repeat, AACATG/ATGTTC (7%) was the most plentiful motif. Other hexameric repeats included AAGCCG/CGGCTT, ACCAGC/CTGGTG, AGAGGG/CCCTCT, and AGGCGG/CCGCCT each accounting for 6%. Additionally, approximately 69% of hexameric repeats were grouped as other types of repeats (**Figure 3F**).

### 3.2 Conservation of SSR in maize and sorghum

To study the evolutionary relationship among sugarcane, maize, and sorghum species and identify unique motifs, we analyzed each motif for the presence of other species. The results showed that sugarcane unigenes were aligned with 11049 unigenes (68.97% of sugarcane unigenes) in sorghum. Of these unigenes, 9382 unigenes were anchored at a single locus, 1002 at two loci, 256 at three loci and four loci, and more for the remaining 409 unigenes. This distribution corresponds to ratios of 84.91%, 9.07%, 2.32%, and 3.70%, respectively. Similarly, 8516 alignments (53.16% of sugarcane unigenes) were revealed between sugarcane and maize; among these unigenes, 4806 mapped to a single locus, 2479 to two loci, 477 to three loci, and 754 unigenes to four loci or more. This distribution corresponds to ratios of 56.43%, 29.11%, 5.60%, and 8.85%, respectively. These results indicate a closer evolutionary relationship between sugarcane and sorghum than that between sugarcane and maize.

### 3.3 Validation and polymorphisms of SSR primers

The results from *in-silico* PCR analysis showed that 235 of 240 SSR primer pairs had potential amplicons in at least one of the three sequenced species including *S. spontaneum*, maize, and sorghum. Interestingly, five of the 242 SSR primer pairs failed to produce potential amplicon in any of these species. Besides, we observed that 9, 13, and 7 SSR primer pairs exclusively generated potential amplicons in the genomes of *S. spontaneum*, maize, and sorghum genome, respectively. Furthermore, 46 SSR primer pairs had potential amplicons in both maize and sorghum genomes, while 9 SSR primer pairs shared potential amplicons in both *S. spontaneum* and sorghum genomes. It is noteworthy that 18 SSR primer pairs were found to have potential amplicons both in *S. spontaneum* and maize genomes. Astonishingly, 133 SSR primer pairs were observed to have potential amplicons in all three genomes (**Supplementary Table S4A**).

Subsequent analysis of the predicted SSR motifs within the potential amplicons generated by SSR primer pairs showed that 219 of 235 SSR primer pairs had the predicted SSR motif in potential amplicons. Of these, 16 SSR primer pairs only existed in both *S. spontaneum* and maize, while 19 SSR primer pairs were found in the sorghum genome. Similarly, 34 SSR primer pairs had SSR motifs present in both maize and sorghum genomes, and 17 SSR primer pairs shared SSR motifs in both *S. spontaneum* and sorghum genomes. Additionally, 21 SSR primer pairs showed SSR motifs in both *S. spontaneum* and maize genomes. In contrast, 106 SSR primer pairs presented SSR motifs in all three genomes (**Supplementary Table S4A**).

Among 235 primer pairs with potential amplicons, 40 SSR primer pairs showed at least one base of the primer sequence that did not match with the amplicon. Further analysis of the binding sites of SSR primer pairs with the potential amplicons showed that 53, 10, and 17 SSR primer pairs fully match with at least one of the potential amplicons in *the S. spontaneum*, maize, and sorghum genome, respectively. Nine SSR primer pairs were found to fully match in both maize and sorghum genomes. Thirty-two SSR primer pairs showed full matches in both *S. spontaneum* and sorghum genomes, and 20 SSR primer pairs fully matched in both *S. spontaneum* and maize genomes. Intriguingly, 54 SSR primer pairs were found to fully match in all three genomes (**Supplementary Table S4A**).

**Figure 4:**
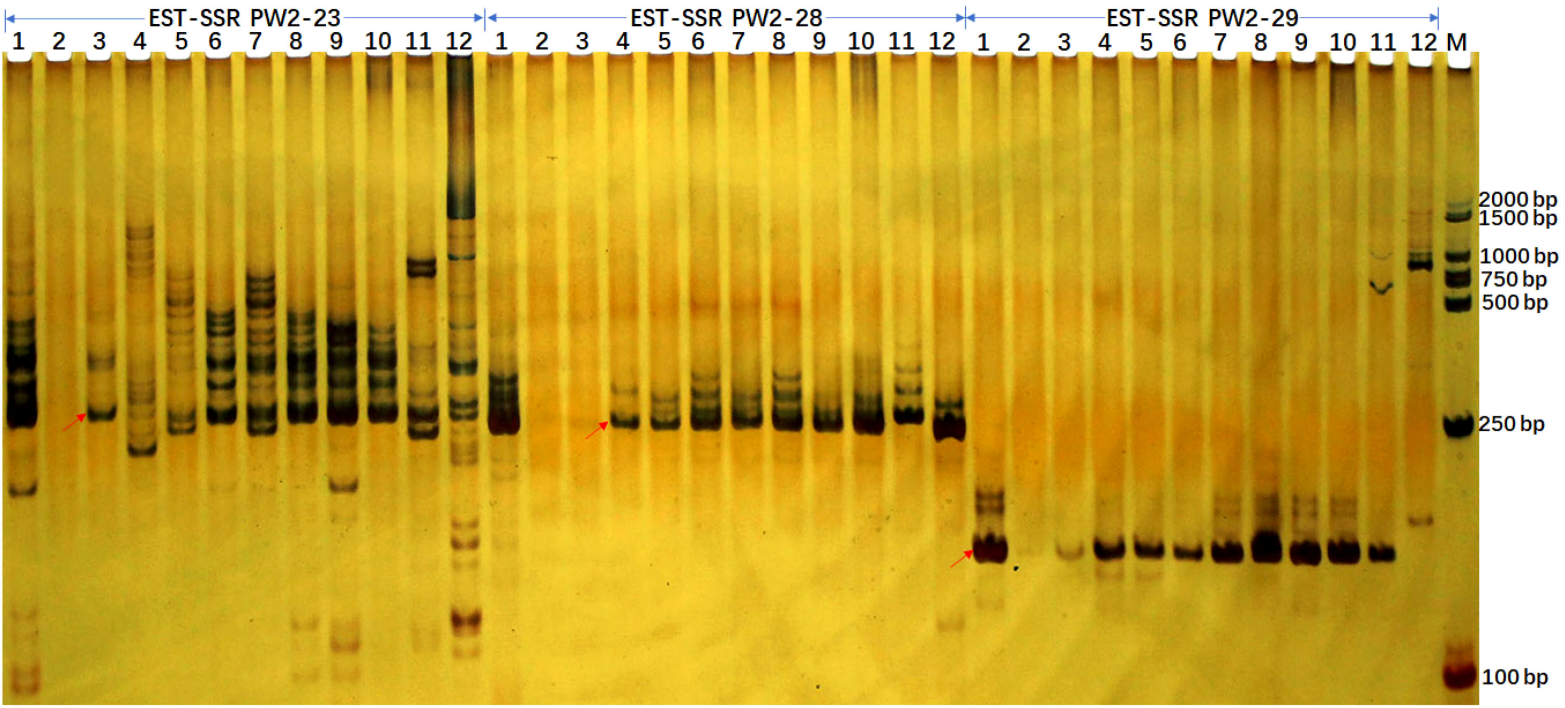
EST-SSR verification profiles of 10 accessions from *Sacchraum*, and single accessions from maize and sorghum each detected by polyacrylamide gel electrophoresis. The EST-SSR profiles with PW2-23, PW2-28, and PW2-29 primer pairs were visualized by silver staining. Lanes 1 to 12 were Yunrui05-782, Yunrui05-767, *S. robustum*, *S. spontaneum*, ROC22, ROC10, Guitang28, Guitang32, Funong40, Funong39, B73 (Maize), Z3116 (Sorghum), respectively. M, BM2000 + 1.5K DNA marker. The arrows show the expected PCR products/potential amplicons.

For the applicability of the deduced SSR markers, we selected 173 primer pairs for the analysis in 10 sugarcane accessions including maize and sorghum using PAGE analysis (**Figure 4**). After optimization, we retained 163 of 173 primers due to clear banding patterns and ease of identification. Among these, 4 were monomeric, 16 were dimeric, 125 were trimeric, 4 were tetrameric, single pentameric, and 3 were hexameric with length ranges spanning from 21 to 109 bp. These 163 SSR loci were capable of amplifying 3-21 alleles within selected accessions, with an average of 9.46 alleles per locus. These SSR markers can be used effectively in genetic diversity analysis, population genetics, and germplasm identification. The polymorphism information content (PIC) values for these SSR loci range from 0.292 to 0.972, with an average PIC value of 0.808, indicating a high level of genetic diversity (**Supplementary Table S4B**).

Additionally, we gained more insights by integrating *in-silico* PCR analysis and amplification of three primer pairs for each locus. We detected expected PCR products containing SSR loci in both *in-silico* PCR analysis and PCR amplification for all three primer pairs in sugarcane. Notably, unexpected PCR bands were amplified for three primer pairs. However, potential amplicons with long fragments, especially more than 1000 bp, were not amplified in maize and sorghum (**Table 4** and **Figure 4**). Additionally, the 265/266 bp bands amplified with primer PW2-23 fully matched in sugarcane and maize were amplified successfully, while the partially matched potential amplicon of 265 bp in sorghum was also amplified. However, for primer pairs PW2-28 and PW2-29, no potential amplicon of the expected PCR products was found in maize and sorghum (**Table 4**). Nonetheless, an almost identical PCR pattern to sugarcane was observed in maize and sorghum (**Figure 4**).

**Table 4:** Potential amplicon analysis results with PW2-23, PW2-28, and PW2-29 primer pairs in sugarcane (*S. spontaneum*), Maize (B73) and Sorghum (Z3116) genome.

Name	SSR motif	Potential amplicon with SSR motif			Potential amplicon without SSR motif		
		*S. spontaneum*	B73	Z3116	*S. spontaneum*	B73	Z3116
PW2-23	(CG)6	265(5)*	**266(4)**	411(3)	None	160	120
		**271(4)**	443(3)			371	126
			522(3)			378	227
			858(3)			522	265
			1139(3)			641	371
			1315(3)			642	955
			1660(3)			647	1211
			1712(5)			690	1370
						837	1749
						1022	1769
						1633	1793
PW2-28	(GAG)5	**253(2)**	737(2)	None	None	209	None
		**255(2)**	738(2)				
		**258(2)**	738(2)				
			738(2)				
			738(2)				
			778(2)				
PW2-29	(CGT)5	**157(5)**	888(2)	1768(2)	None	60	131
		**158(5)**	1200(2)			60	335
		158(5)	1991(2)			328	744
							1961

*: 265(5) represents the size of potential amplicon is 265 bp and 5 SSR motif copies exist in amplicon, respectively. Bold means primer pair fully match with binding site.

### 3.4 Functional annotation of sugarcane unigenes harboring the SSRs

**Figure 5:**
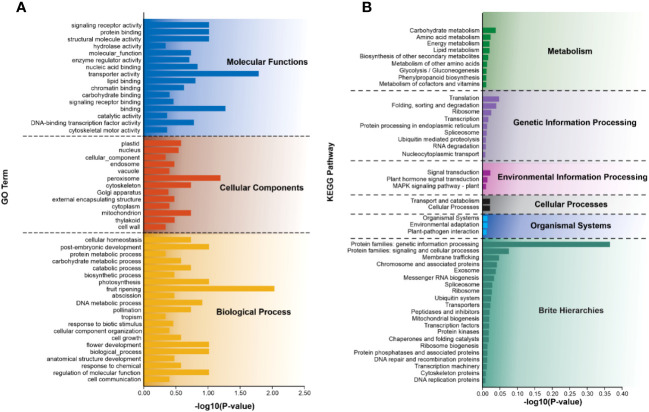
Summary of functional annotation of SSR-containing unigenes. **(A)** GO and **(B)** KEGG represent different classes based on the predicted function of the top 50 SSR-containing unigenes. The y-axis indicates the number of genes in each specific category.

To explore the potential functions of SSR-containing unigenes, all of these unigenes were annotated against the publicly available functional databases. This analysis indicated that 38.75% of unigenes were associated with GO, while 43.96% were linked to the KEGG. These SSR-containing unigenes were further classified into three major GO functional categories including, biological process, cellular component, and molecular function (**Figure 5A** and **Supplementary Table S5A**). Within biological processes, unigenes related to post-embryonic development, photosynthesis, fruit ripening, DNA metabolic process, flower development, and regulation of molecular function accounted for the largest proportion. The cellular component category primarily represented unigenes involved in peroxisome, cytoskeleton, and mitochondrion. In the molecular function category, the most enriched unigenes were involved in signaling receptor activity, protein binding, structural molecule activity, and transporter activity binding.

Furthermore, these unigenes annotated 195 KEGG metabolism pathways, which were classified into six categories including cellular processes, environmental information processing, genetic information processing, metabolism, organismal systems, and brite hierarchies (**Figure 5B**). In the second level of the pathway classification, prominent categories included carbohydrate metabolism, translation, signal transduction, transport and catabolism, environmental adaptation, protein families: genetic information processing, and protein families associated with signaling and cellular processes. Additional details of each category are provided in **Figure 5B** and **Supplementary Table S5B**.

### 3.5 Genetic diversity and relationships among genotypes

To explore the genetic similarity of sugarcane accession, we conducted a cluster analysis based on a matrix for the presence and absence of deduced alleles. **Figure 5** represents the clustering results in the form of phylogenetic trees. The phylogenetic clustering unveiled four distinct accession clusters: “*S. robustum*”, “Yunrui05-767”, “ROC10”, “ROC22”, “Guatang28”, and “Guatang32” form a major cluster; Cluster-I, “Funong40”, and “Funong39” are present in Cluster-II. “Yunrui05-782” and “*S. spontaneum*” formed a separate cluster each (Cluster-III and IV) at the bottom of the phylogenetic tree (**Figure 5**). The accession in Cluster-I shares a genetic distance value of 7.4 in relation to other accessions in Cluster-II. Notably, the Taiwan accessions, “ROC22” and “ROC10”, as well as the Fujian varieties, “Funong40” and “Funong39” showed a genetic distance of 2.5 between them, indicating a higher degree of similarity as determined by the studied SSR markers. The largest genetic distances were recorded between Yunnan varieties clustered in different clades.

## 4 Discussion

SSRs are known for their repeatability and polymorphism, extensively being used in unveiling the genetic diversity and markers of assisted breeding programs (Wang et al., 2010) of various plant species including cucumber, cotton, foxtail millet, rice, citrus, horse gram, maize, and sweet cane (Zhou et al., 2021). However, the application of EST-SSR markers has been limited in Sugarcane (*Saccharum* spp.) (Zhang et al., 2012). For instance, Xiao et al. (2020) identified a set of 349 EST-SSR markers. In this study, we have significantly expanded these marker resources by developing a novel set of 4960 EST-SSR markers. Among these EST-SSRs, 163 primer pairs proved effective for identifying 10 sugarcane accessions, demonstrating the suitability of transcriptome sequences as valuable resources for SSR markers’ development.

**Figure 6:**
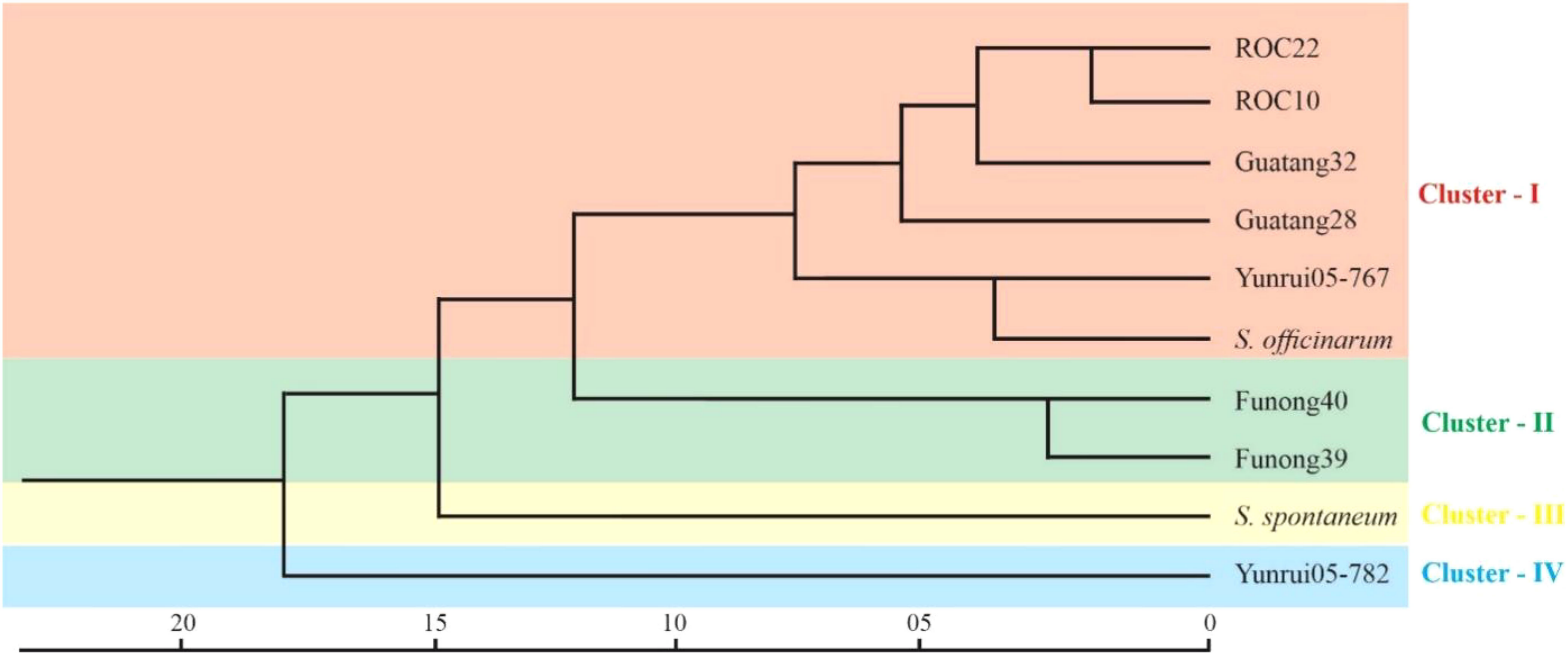
Cluster analysis of Sugarcane accession by SSR markers. The scale at the bottom represents the genetic distance between all the accessions.

The cluster results aligned well with the origin and pedigrees of 10 sugarcane accessions, providing insights into their relationships (**Figure 6** and **Table 1**). For instance, sugarcane accessions from Fujian, Guangxi, and Taiwan clustered according to their breeding regions, while those with common parents clustered together. Additionally, our analysis revealed that *S. officinarum* shared a closer relationship with cultivated sugarcane compared to *S. spontaneum*. Interestingly, two cultivated sugarcane lines (Yunrui05-782 and Yunrui05-767) derived from hybrid wild species were distinct from other cultivated sugarcane varieties, highlighting the potential of wild species in expanding the genetic basis of cultivated sugarcane through sexual hybridization. We also explored the distribution of SSRs within the genomes of 10 sugarcane cultivars, observing a relatively high frequency of SSRs, approximately 1/4.43 kb. This frequency is comparable to certain other plant species such as *P. violascens* (1/4.45 kb), Chinese cabbage (1/4.67 kb), and Wheat (1/5.46 kb) but significantly higher than in Arabidopsis (1/13.83 kb) (Cardle et al., 2000; Peng and Lapitan, 2005; You et al., 2015; Cai et al., 2019). The types of repeat motifs in this study were not uniformly distributed in the sugarcane genome. In general, unlike former research studies on sugarcane (**Table 4**) by Singh et al. (2013); Xiao et al. (2020); Ukoskit et al. (2012), and Ul Haq et al. (2016), we found that the monomeric repeats accounted for the largest proportion, at 44.33% followed by tetrameric and dimeric repeats which were 39.68% and 13.10%, respectively (**Table 3**). These results are different from Xiao et al. (2020) in which trimeric repeats were most abundant. Dimeric and trimeric repeats were predominant when excluding monomeric repeats. Additionally, we found that the proportion of tetrameric, pentameric, and hexameric repeats was significantly lower than those reported by Xiao et al. [16] and other species (**Table 5**). Overall, our findings contribute to a deeper understanding of the SSR landscape in sugarcane and its implications for genetic studies and breeding programs.

**Table 5:** Comparison of frequency of microsatellites of different species.

Plant	Sugarcane*	Sugarcane**	Arabidopsis†	*Triticum aestivum*†	*Dendrocalamus latiflorus*†	*Phyllostachys violascens*†
Di-	23.55	22.06	26.27	20.77	16.1	48.06
Tri-	71.30	29.90	73.04	74.26	47.7	48.84
Tetra-	2.97	9.51	0.72	3.36	26.1	2.54
Penta-	1.05	24.03	0	1.12	6.9	0.42
Hexa-	1.15	15.48	0	0.5	3.3	0.14
Total	2761	37055	1070	43598	22305	9257

*this study; **Xiao et al., 2020; †Cai et al., 2019 all values in percent % except total number of SSR markers.

As shown in **Figure 2**, the A/T motif was the predominant monomeric repeat (88%). In contrast, the GC/CT repeats accounted for 56%, which was higher than what was reported by Xiao et al. (2020) in sugarcane. Additionally, the abundance exceeded in other species such as taro (52.86%) (You et al., 2015), pigeon pea (16.7%) (Dutta et al., 2011), and wheat (8.7%) (Peng and Lapitan, 2005). Of trimeric repeats, CCG/CGG was the most predominant (48%), higher than the previous findings in taro (You et al., 2015), sugarcane (4.84%) (Xiao et al., 2020), and rice and maize (Cardle et al., 2000). The CGC/GCG trimeric repeat at 17% was the second most abundant, which was lower than in *P. violascens* (3.45%) (Cai et al., 2019) and sugarcane (4.74%) (Xiao et al., 2020). The prevalence of trimeric repeat, CCG/CGG, a characteristic trimeric repeat in monocots was verified by our results but was rare in dicotyledonous plants (You et al., 2015; Cai et al., 2019; Xiao et al., 2020). The PIC is a critical metric in assessing the level of polymorphism of SSR markers, with a PIC value greater than 0.5 indicating a high level of polymorphism (Botstein et al., 1980). In our study, based on 163 EST-SSR markers, PIC values ranged from 0.292 to 0.972 with an average PIC value of 0.809 (**Table S4**). These findings align with Xiao et al. (2020) (0.70–0.94), Singh et al. (Singh et al., 2013) (0.12–0.99; 0.85), and Ul Haq et al. (2016) (0.51–0.93; 0.83).

In general, EST-SSR primer pairs and corresponding SSR loci were designed and aligned in *S. spontaneum*, sorghum, and maize in this study, which provided a possible way to develop EST-SSRs for sugarcane breeders. First, we developed EST-SSRs using sugarcane ES sequences or functional genes in the sugarcane genome. Some of the EST-SSR primer pairs were synthesized and successfully amplified by PCR in 10 sugarcane cultivars with sorghum and maize. Interestingly, our analysis revealed that a subset of SSR primer pairs (9 in *S. spontaneum*, 13 in maize, and 7 in sorghum) produced potential amplicons exclusively in one of these genomes. This observation suggests that while these species share some genetic similarities, they have also undergone unique evolutionary processes that have led to the development of distinct SSR loci. Such species-specific SSR markers can serve as important indicators of genetic divergence and could shed light on the evolutionary history of these species.

In sunflowers, most SSR-containing genes are involved in various biological processes such as cellular and metabolic processes (Lulin et al., 2012). Parmar et al., (Parmar et al., 2022) reported that most of the SRR-containing genes are involved in biological regulation and metabolic processes, which is consistent with the present study. The most important molecular functions of the GO-enriched genes in the present study are transport activity, binding, signaling receptor activity, protein activity, and catalytic activity. Additionally, the key biological processes associated with GO enrichment genes include fruit ripening, post-embryonic development, photosynthesis, and regulation of molecular functions. KEGG analysis of SSR-containing genes showed an important metabolic pathway such as carbohydrate metabolism and amino acid metabolism. The genetic information processing category was the second largest group.

## 5 Conclusion

In the present study, we achieved several significant outcomes. We successfully aligned sugarcane unigenes with sorghum and maize, leading to the identification and development of a valuable set of EST-SSR markers in sugarcane. A total of 4960 potential SSR markers were identified and of 240 randomly selected primer pairs, 173 were assessed for polymorphism. Among these, 163 primer pairs exhibited polymorphism when applied to 10 sugarcane accessions. Furthermore, we annotated 4203 SSR-containing unigenes into GO and KEGG databases, shedding light on their potential functions and pathways. Notably, we found that 56.43% of sugarcane unigenes mapped in maize genome to a single locus, 29.11% at two loci, 5.6% at three loci, and 8.58% with other loci. This suggests a distinct evolutionary relationship between sugarcane and sorghum with more duplication events occurring in maize chromosome segments. We believe these results have broad implications, contributing an important resource for future genomic and genetic studies in sugarcane but also serving as a powerful tool for studying evolutionary adaptation and genetic relationships in other related species.

## Data availability statement

The original contributions presented in the study are included in the article/**Supplementary Material**. Further inquiries can be directed to the corresponding authors.

## Ethics statement

This article does not contain any studies with human participants or animals performed by any of the authors.

## Author contributions

YL and PL: Conceptualization and Experimental Design, HJ: Data Collection, HJ and MW: Data Curation, YW and XZ: Resources—Plant materials Preparation, HJ and MW: drafted the manuscript, YL, SB, and PL: Review and Editing the drafted manuscript. All authors contributed to the article and approved the submitted version.
